# Cooperative 2′-O-methylation of the wobble cytidine of human elongator tRNA^Met^(CAT) by a nucleolar and a Cajal body-specific box C/D RNP

**DOI:** 10.1101/gad.326363.119

**Published:** 2019-07-01

**Authors:** Patrice Vitali, Tamás Kiss

**Affiliations:** 1 Laboratoire de Biologie Moléculaire Eucaryote, UMR5099, Centre National de la Recherche Scientifique, Centre de Biologie Intégrative, Université Paul Sabatier, 31062 Toulouse Cedex 9, France;; 2 Biological Research Centre, Hungarian Academy of Sciences, 6726 Szeged, Hungary

**Keywords:** box C/D small nucleolar RNA, small Cajal body RNA, 2′-O-methylation, tRNA-derived fragment, angiogenin

## Abstract

Site-specific 2′-O-ribose methylation of mammalian rRNAs and RNA polymerase II-synthesized spliceosomal small nuclear RNAs (snRNAs) is mediated by small nucleolar and small Cajal body (CB)-specific box C/D ribonucleoprotein particles (RNPs) in the nucleolus and the nucleoplasmic CBs, respectively. Here, we demonstrate that 2′-O-methylation of the C34 wobble cytidine of human elongator tRNA^Met^(CAT) is achieved by collaboration of a nucleolar and a CB-specific box C/D RNP carrying the SNORD97 and SCARNA97 box C/D 2′-O-methylation guide RNAs. Methylation of C34 prevents site-specific cleavage of tRNA^Met^(CAT) by the stress-induced endoribonuclease angiogenin, implicating box C/D guide RNPs in controlling stress-responsive production of putative regulatory tRNA fragments.

## Abstract (precis)

In this study, Vitali et al. demonstrate that 2′-O-methylation of the C34 wobble cytidine of human elongator tRNA^Met^(CAT) is achieved by collaboration of a nucleolar and a CB-specific box C/D RNP carrying the SNORD97 and SCARNA97 box C/D 2′-O-methylation guide RNAs.

Posttranscriptional modification of selected ribonucleotides is an important step of the biogenesis of cellular RNAs (Grosjean 2005; Boccaletto et al. 2018). Mature tRNAs are heavily decorated by various types of covalent nucleotide modifications that support their correct folding and promote efficient and faithful tRNA aminoacylation and anticodon–codon interaction (Duechler et al. 2016). Modifications at the tRNA anticodon loop, especially at the wobble position 34, are important for accurate decoding at the A site of the ribosome (Grosjean et al. 2010; Jackman and Alfonzo 2013).

In response to stress stimuli, cytoplasmic tRNAs are frequently cleaved by the anticodon targeting endoribonuclease angiogenin. The resulting tRNA-derived fragments (tRFs) have been proposed to function as important regulatory RNAs controlling stress signaling, adaptive translation, and mRNA decay (Yamasaki et al. 2009; Ivanov et al. 2011; Goodarzi et al. 2015). Stress-induced tRF production can be controlled by tRNA modifications altering angiogenin activity (Lyons et al. 2018). For instance, conversion of the wobble guanosine to queuosine protects human tRNAs His and Asn from angiogenin cleavage (Wang et al. 2018). Cytosine 5 methylation of *Drosophila* and mouse tRNAs Asp, Val, and Gly at C38 as well as mouse and human tRNAs Asp, Glu, Gly, His, Lys, and Val at the wobble cytidine inhibits angiogenin-mediated cleavages of these tRNAs (Goll et al. 2006; Schaefer et al. 2010; Tuorto et al. 2012; Blanco et al. 2014).

The most common site-specific RNA modification reactions (namely, 2′-O-ribose methylation of the four ribonucleotides and conversion of uridines into pseudouridine) are frequently catalyzed by evolutionarily conserved box C/D 2′-O-methylation and box H/ACA pseudouridylation and guide ribonucleoprotein particles (RNPs) (Watkins and Bohnsack 2012). In unicellular archaea, box C/D and H/ACA RNPs direct 2′-O-methylation and pseudouridylation of rRNAs and tRNAs (Omer et al. 2000; Dennis and Omer 2005). In eukaryotic cells, box C/D and H/ACA guide RNPs accumulate in either the nucleolus or the nucleoplasmic CBs and, accordingly, are called small nucleolar RNPs (snoRNPs) or small CB RNPs (scaRNPs) (Darzacq et al. 2002). While snoRNPs mediate 2′-O-methylation and pseudouridylation of rRNAs, scaRNPs direct modification of RNA polymerase II (RNAPII) transcribed spliceosomal small nuclear RNAs (snRNAs) (Darzacq et al. 2002; Jády et al. 2003). Each guide RNP is composed of a guide RNA and four RNP proteins, including the 2′-O-methyltransferase and pseudouridine synthase (Watkins and Bohnsack 2012). The box C/D 2′-O-methylation guide RNAs carry the conserved 5′-terminal C (RUGAUGA) and 3′-terminal D (CUGA) boxes and their usually imperfect internal copies: the C′ and D′ boxes. The target recognition or antisense elements immediately precede the D and/or D′ boxes, and their fifth nucleotide counted from the D or D′ box faces the substrate nucleotide selected for 2′-O-methylation (Kiss-László et al. 1996). Besides the methyltransferase fibrillarin, each box C/D RNA associates with the Nop56, Nop58, and 15.5-kDa box C/D RNP proteins. The box C/D and H/ACA scaRNAs carry specific CB localization signal sequences that, through interacting with CB proteins such as WDR79, target scaRNPs into the CB (Richard et al. 2003; Tycowski et al. 2009; Marnef et al. 2014).

Mammalian cells also express many C/D and H/ACA RNAs lacking rRNA- or snRNA-specific antisense elements. The function of these “orphan” snoRNAs is unknown. Here, we demonstrate that the previously characterized human SNORD97 orphan box C/D snoRNA and its CB-specific functional homolog, termed SCARNA97, direct 2′-O-methylation of the wobble cytidine C34 of human elongator tRNA^Met^(CAT) in a cooperative fashion. We also show that 2′-O-methylation of C34 protects tRNA^Met^(CAT) from endonucleolytic cleavage by stress-induced angiogenin.

## Results and Discussion

### Differential subnuclear localization of two isoforms of human SNORD97

**Figure 1.**
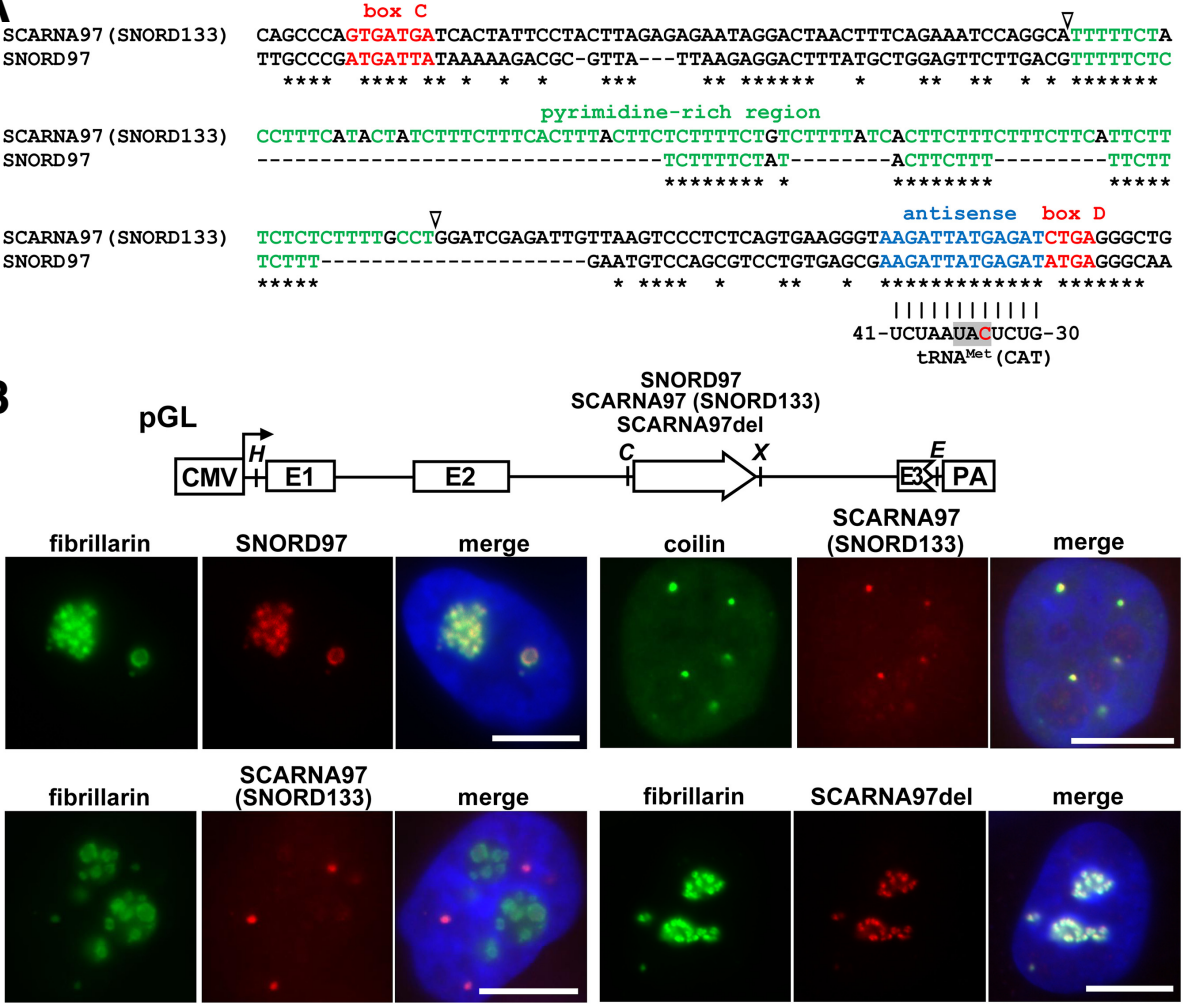
Characterization of human SNORD97 and SCARNA97 (SNORD133). (*A*) Alignment of SNORD97 and SCARNA97 sequences. The C and D boxes (red), putative antisense elements (blue), and pyrimidine residues in the internal regions (green) are highlighted. Asterisks mark identical nucleotides. Predicted base-pairing with tRNA^Met^(CAT) is shown. The anticodon (shaded) and the wobble cytidine (red) are highlighted. Open arrowheads indicate the 5′ and 3′ ends of the internal deletion in SCARNA97del. (*B*) In situ localization of transiently overexpressed SNORD97 and SCARNA97. Schematic structure of the pGL expression construct is shown. The cytomegalovirus (CMV) promoter with the transcription start site (arrow), the exons (E1, E2, and E3), and the polyadenylation site (PA) of the human β-globin gene are shown. The open arrow indicates the *SNORD97*, *SCARNA97* (*SNORD133*), and *SCARNA97del* test RNA genes. Relevant restriction sites are shown. (H) HindIII; (C) ClaI; (X) XhoI. Subnuclear distribution of SNORD97, SCARNA97, and SCARNA97del was determined by fluorescent in situ hybridization (FISH). Nucleoli were visualized by transient expression of GFP-fibrillarin. CBs and nuclear DNA were stained with anticoilin and DAPI, respectively. Scale bar, 10 µm.

Previous analysis of human WDR79-associated RNAs discovered Alu-derived H/ACA RNAs and detected novel classical snoRNAs (Jády et al. 2012). Among the new box C/D RNAs, we identified a 216-nucleotide (nt)-long RNA that, during the course of this study, was also detected in fibrillarin and coilin interactomes and was termed SNORD133 (Kishore et al. 2013; Machyna et al. 2014). Northern blot analysis confirmed expression of SNORD133 in human HeLa cells (Supplemental Fig. S1). We noticed that SNORD133, especially its D box-dependent putative target recognition region, shows strong sequence similarity to the previously characterized human SNORD97, suggesting that these two RNAs represent functional isoforms (Fig. 1A; Vitali et al. 2003).

**Figure 2.**
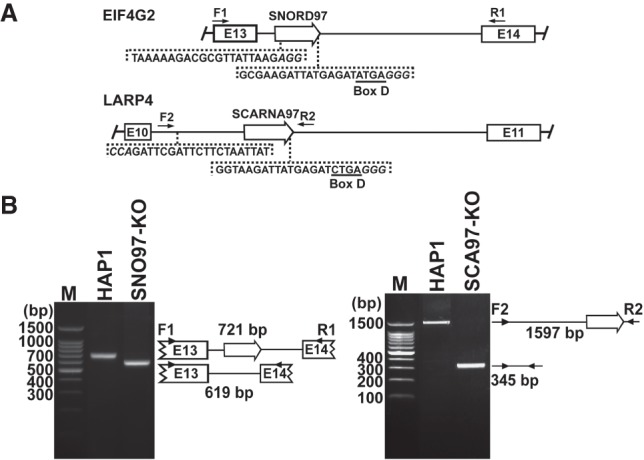
Construction of *SNORD97* knockout and *SCARNA97* knockout HAP1 cells. (*A*) Strategy for CRISPR–Cas9-mediated excision of *SNORD97* and *SCARNA97* genes. The intronic *SNORD97* and *SCARNA97* genes (open arrows), the neighboring exons of the *EIF4G2* (E13 and E14) and *LARP4* (E10 and E11) host genes, and the forward (F1 and F2) and reverse (R1 and R2) primers used for PCR analyses are shown. The genomic sequences targeted by sgRNAs are indicated in sense orientation. The PAM (or complementary) sequences are in italics. The D boxes are underlined. (*B*) PCR analyses of genomic DNAs obtained from *SNORD97* knockout (SNO97-KO) and *SCARNA97* knockout (SCA97-KO) cells. The amplified DNAs were analyzed on 2% agarose gels. Structures and expected sizes of the amplified genomic fragments are shown. (Lane *M*) DNA size marker in base pairs.

Previous cell fractionation experiments localized SNORD97 to the nucleolus (Vitali et al. 2003). SNORD133, however, has been detected among RNAs coimmunopurified with the CB proteins WDR79 and coilin, suggesting that it may accumulate in the CB (Jády et al. 2012; Machyna et al. 2014). To settle this inconsistency, we determined the subcellular localization of both SNORD97 and SNORD133 with fluorescent in situ hybridization (FISH) microscopy (Fig. 1B). The human *SNORD97* and *SNORD133* genes are located within intron regions of the *EIF4G2* and *LARP4* genes in sense orientations, confirming that SNORD97 and SNORD133 are bona fide intron-encoded RNAs (see Fig. 2A). To facilitate FISH detection, the two RNAs were transiently overexpressed in HeLa cells by using the pGL intronic snoRNA expression plasmid (Fig. 1B; Darzacq et al. 2002). Cells were hybridized with fluorescently labeled oligonucleotides specific for SNORD97 or SNORD133 and immunostained with antibodies against the nucleolar fibrillarin and CB-specific coilin. Consistent with previous results, SNORD97 showed a nucleolar colocalization with fibrillarin. In contrast, the SNORD133 probe highlighted two to eight nucleoplasmic dots, which, upon counterstaining the cells with anticoilin antibody, proved to be CBs. Thus, contrary to their sequence similarity, SNORD97 and SNORD133 possess differential subnuclear localizations. While SNORD97 is an snoRNA, SNORD133 accumulates in CBs and therefore has been renamed SCARNA97.

Vertebrate box C/D scaRNAs carry internal G.U/U.G wobble stems that function as CB localization signals (Marnef et al. 2014). SCARNA97, however, lacks GU repeats but carries a 93-nt-long pyrimidine-rich internal sequence (Fig. 1A; Supplemental Fig. S2). So far, no similar pyrimidine-dense region has been detected in other small RNAs, including snRNAs, snoRNAs, and scaRNAs. To test the potential importance of the pyrimidine-rich stretch of SCARNA97 in CB localization, a truncated SCARNA97 (SCARNA97del) lacking the U65–U154 internal sequences was expressed in HeLa cells. SCARNA97del concentrated in the nucleolus together with fibrillarin, demonstrating that the pyrimidine-rich region is essential for targeting SCARNA97 into the CB (Fig. 1B). Thus, we concluded that SCARNA97 carries a novel box C/D scaRNA-specific CB localization signal distinct from the previously identified G.U/U.G wobble stem (Marnef et al. 2014).

### SNORD97 and SCARNA97 are predicted to direct 2′-O-methylation of elongator tRNA^Met^(CAT)

A systematic blast search identified apparent homologs for both *SNORD97* and *SCARNA97* in all vertebrate genomes investigated (Supplemental Fig. S3A,B), but only one common *SNORD97/SCARNA97* gene candidate could be detected in invertebrates, including insects (*Drosophila*, silkworms, and honey bees), flowering plants (*Arabidopsis* and rape), and tunicates (*Ciona intestinalis*) (Supplemental Fig. S3C). This suggests that vertebrate *SNORD97* and *SCARNA97* genes originated from a gene duplication event at the early stage of vertebrate evolution. Apart from bird SNORD97, all SNORD97 and SCARNA97 candidates carry phylogenetically invariant D box-dependent putative target recognition motifs, suggesting that they direct 2′-O-methylation of an evolutionarily conserved RNA. Bioinformatic analysis of stable cellular RNAs, such as rRNAs, snRNAs, snoRNAs, scaRNAs, tRNAs, and microRNAs, identified the elongator tRNA^Met^(CAT) as a possible target for SNORD97 and SCARNA97 (Fig. 1A). The antisense elements of SNORD97 and SCARNA97 are predicted to select the C34 wobble cytidine of tRNA^Met^(CAT) for 2′-O-methylation. The anticodon stem–loop regions of vertebrate, insect, and plant elongator tRNAs Met(CAT) are highly conserved and can base-pair to the antisense elements of SNORD97 and SCARNA97 to position their wobble cytidines for 2′-O-methylation (Supplemental Fig. S4). Finally, in archaeal and vertebrate elongator tRNAs Met(CAT), the wobble cytidine is 2′-O-methylated, and, in Archaea, this methyltranfer reaction is catalyzed by box C/D RNPs (Joardar et al. 2011; Boccaletto et al. 2018).

### Human SNORD97 and SCARNA97 cooperate in 2′-O-methylation of tRNA^Met^(CAT)

To investigate SNORD97 and SCARNA97 participation in 2′-O-methylation of elongator tRNA^Met^(CAT), expression of either RNAs was knocked out in human HAP1 cells. Internal fragments of the 13th and 10th introns of the *EIF4G2* and *LARP4* genes encompassing the *SNORD97* and *SCARNA97* genes were deleted by CRISPR–Cas9 genome editing (Fig. 2A). The accuracy of *SNORD97* and *SCARNA97* gene excisions was verified by PCR amplification of the truncated *EIF4G2* and *LARP4* gene fragments (Fig. 2B). Likewise, correct processing and efficient accumulation of the EIF4G2 and LARP4 mRNAs in the CRISPR–Cas9-treated cells were verified by RT-PCR (Supplemental Fig. S5).

**Figure 3.**
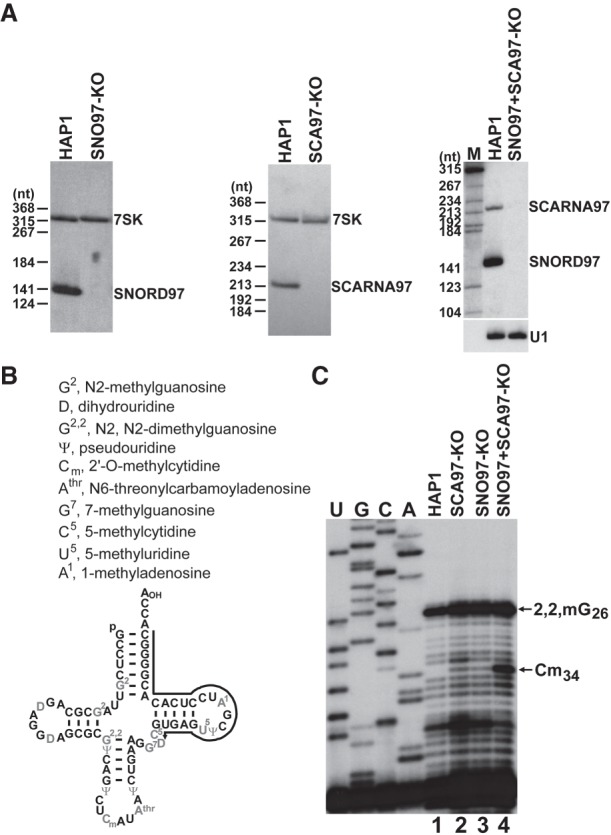
Both SNORD97 and SCARNA97 participate in 2′-O-methylation of tRNA^Met^(CAT). (*A*) Northern blot analysis of SNORD97 and SCARNA97 expression in HAP1, *SNORD97* knockout (SNO97-KO), *SCARNA97* knockout (SCA97-KO), and *SNORD97 + SCARNA97* knockout (SNO97 + SCA97-KO) cells. The 7SK and U1 snRNAs are positive controls. (*B*) Two-dimensional structure of human elongator tRNA^Met^(CAT) with modified nucleotides (Boccaletto et al. 2018). Position of the oligonucleotide used for primer extension is shown. (*C*) Mapping of 2′-O-methylation of tRNA^Met^(CAT). Cellular RNAs extracted from the indicated cell lines were subjected to partial alkaline hydrolysis. Terminally labeled oligonucleotide primer was annealed to each RNA sample and extended with reverse transcriptase. (Lanes *U*,*G*,*C*,*A*) Dideoxy sequencing ladders.

Northern analysis failed to detect accumulation of SNORD97 and SCARNA97 in the *SNORD97* knockout and *SCARNA97* knockout cells, while expression of the 7SK snRNA was not affected (Fig. 3A). The 2′-O-methylation state of elongator tRNA^Met^(CAT) at C34 was monitored by primer extension analysis performed on partially alkaline hydrolyzed cellular RNAs with a 5′ terminally labeled oligonucleotide primer complementary to the 3′-terminal part of tRNA^Met^(CAT) (Fig. 3B,C). Since 2′-O-methyl groups confer resistance to alkaline hydrolysis, the 2′-O-methylated nucleotides appear as gaps in the ladder of primer extension products (Kiss-László et al. 1996). Mapping of tRNA^Met^(CAT) derived from HAP1 control cells resulted in a gap 1 nt before C34, confirming its positive 2′-O-methylation status (Fig. 3C, lane 1). When tRNAs from SNORD97 knockout or SCARNA97 knockout cells were mapped, primer extension stop signals appeared in the gap corresponding to Cm34, indicating that 2′-O-methylation of C34 is at least partially inhibited in the absence of SNORD97 or SCARNA97 (Fig. 3C, lanes 2,3).

Using the same genome manipulation strategies depicted in Figure 2A, we next created a HAP1 double-knockout cell line devoid of both *SNORD97* and *SCARNA97* genes. The *SNORD97 + SCARNA97* knockout cells failed to accumulate SNORD97 and SCARNA97 but expressed the U1 snRNA (Fig. 3A). Primer extension mapping of partially hydrolyzed tRNA^Met^(CAT) resulted in a very intense stop at C34, suggesting that simultaneous elimination of SNORD97 and SCARNA97 fully abolished 2′-O-methylation of C34 (Fig. 3C, lane 4). We concluded that both SNORD97 and SCARNA97 are required for efficient 2′-O-methylation of the wobble cytidine of human tRNA^Met^(CAT).

### SNORD97 and SCARNA97 expression restores 2′-O-methylation of tRNA^Met^(CAT)

**Figure 4.**
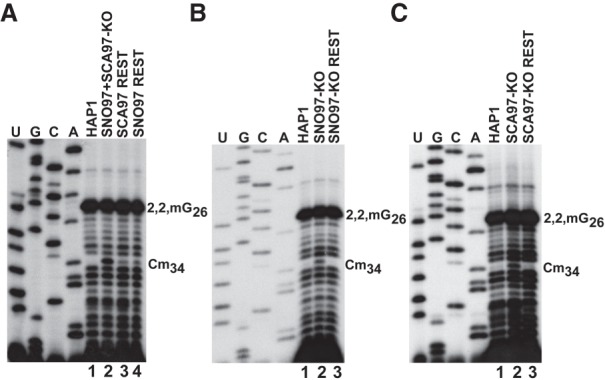
Primer extension mapping of 2′-O-methylation of tRNA^Met^(CAT) obtained from the indicated cell lines before and after restoration (rest) of SNORD97 or SCARNA97 expression. For other details, see Figure 3.

To rule out the formal possibility that defective 2′-O-methylation of tRNA^Met^(CAT) in SNORD97 knockout and/or SCARNA97 knockout cells was due to undesired off-target activity of CRISPR–Cas9, we restored SNORD97 and SCARNA97 expression in these cells (Supplemental Fig. S6). As demonstrated above, the ectopically expressed SNORD97 and SCARNA97 concentrated in the nucleolus and CB, respectively (see Fig. 1B; data not shown). Stable expression of either SNORD97 or SCARNA97 in the *SNORD97 + SCARNA97* knockout cells largely reduced the primer extension stop signals at C34, indicating that SNORD97 or SCARNA97 accumulation partially restored C34 methylation of tRNA^Met^ (CAT) (Fig. 4A, cf. lanes 3,4, and 2). Reinstatement of SNORD97 accumulation in *SNORD97* knockout cells or SCARNA97 expression in *SCARNA97* knockout cells completely eliminated reverse transcriptase stops at C34, demonstrating that 2′-O-methylation of C34 was fully restored (Fig. 4B,C, lane 3). Thus, we propose that 2′-O-methylation of the wobble cytidine of human tRNA^Met^ (CAT) is cooperatively accomplished by the nucleolar SNORD97 and the CB-specific SCARNA97 RNPs.

Demonstration that human SNORD97 and SCARNA97 direct 2′-O-methylation of tRNA^Met^(CAT) provides the first experimental evidence to the long-lived hypothesis that orphan snoRNPs—or at least some of them—function in modification of cellular RNAs different from rRNAs and snRNAs. Contrary to their common target RNA, SNORD97 and SCARNA97 accumulate in two distinct subnuclear compartments, the nucleolus and CB, which provide the nuclear locale for posttranscriptional processing and modification of rRNAs and spliceosomal snRNAs, respectively (Jády et al. 2003). Consistent with this, snoRNPs directing rRNA modifications are restricted to the nucleolus, while RNPs guiding 2′-O-methylation and pseudouridylation of RNAPII-synthesized spliceosomal snRNAs are localized to the CB (Darzacq et al. 2002). These clear waters were first muddied by detection of guide RNAs specific for the RNAPIII transcribed U6 spliceosomal snRNA in either the nucleolus or the CB, raising the possibility that some U6 modifications occur in the nucleolus, while others take place in the CB (Tycowski et al. 1998, 2009; Ganot et al. 1999; Deryusheva and Gall 2013). Ribose methylation of C34 in tRNA^Met^(CAT) is the first RNA-guided RNA modification reaction that requires both a nucleolar and a CB-specific guide RNP, raising the possibility that this modification reaction takes place partly in the nucleolus and CB. The human tRNA^Met^(CAT) is transcribed from at least nine closely related genes (Supplemental Fig. S4). In theory, it is conceivable that some tRNA^Met^(CAT) transcripts cycle through the nucleolus, while others transit through the CB to undergo snoRNP- or scaRNP-mediated 2'-O-methylation. However, our FISH analyses failed to detect mature or precursor tRNA^Met^(CAT) sequences in the nucleolus and CB (data not shown). This may indicate that maturing tRNA^Met^(CAT) transits through the nucleolus and CB very rapidly or is simply excluded from these nuclear structures. Concerning the second scenario, it is possible that a fraction of SNORD97 and/or SCARNA97 localizes to the nucleoplasm and directs 2′-O-methylation of tRNA^Met^(CAT). This idea seems to be supported by the observation that scaRNPs can efficiently modify spliceosomal snRNAs in mammalian cells lacking CBs (Deryusheva et al. 2012). Thus, dissection of the precise role of the nucleolus and CB in 2′-O-methylation of tRNA^Met^(CAT) requires further efforts.

### SNORD97- and SCARNA97-directed 2′-O-methylation protects tRNA^Met^(CAT) from angiogenin cleavage under arsenite stress

**Figure 5.**
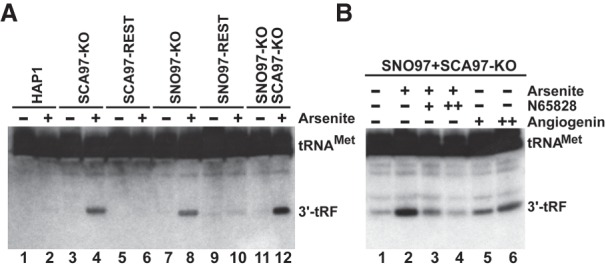
Expression of SNORD97 and SCARNA97 inhibits angiogenin cleavage of tRNA^Met^(CAT). (*A*) Arsenite-induced oxidative stress promotes processing of tRNA^Met^(CAT) into stable 3′ tRF in the absence of SNORD97 and/or SCARNA97. Human cells indicated *above* were incubated in either the presence or absence of 250 μM arsenite for 2 h before RNA extraction. Accumulation of 3′ tRF was monitored by Northern blotting. (*B*) Angiogenin mediates cleavage of tRNA^Met^ (CAT). *SNORD97 + SCARNA97* knockout cells either treated with arsenite or nontreated were further incubated for 2 h in a medium supplemented with 40 or 96 µM angionenin inhibitor N-65828 or 50 or 100 ng/mL recombinant angiogenin as indicated. RNAs were extracted from each cell line and analyzed by Northern blotting.

In response to stress stimuli, the tRNA anticodon loops are frequently cleaved by the stress-induced endoribonuclease angiogenin to produce stable 5′ or 3′ tRFs, which can function as important regulatory RNAs (see above). Angiogenin preferentially cuts between C and A residues. In the absence of SNORD97 and SCARNA97, we observed unexpectedly strong primer extension stops 1 nt before the C34 residue of tRNA^Met^(CAT) ( see Fig. 3C, lane 4). To test whether these intense stops are due to partial angiogenin cleavage of tRNA^Met^(CAT) between C34 and A35, *SNORD97* knockout, *SCARNA97* knockout, *SNORD97 + SCARNA97* knockout, and control HAP1 cells were treated with arsenite to induce oxidative stress and activate angiogenin (Fig. 5A). Northern blot analyses detected strong 3′ tRF accumulations in arsenite-treated cells lacking SNORD97 and/or SCARNA97 (Fig. 5A, lanes 4,8,12). The 5′ tRF of tRNA^Met^(CAT) was not detectable, indicating that it is rapidly degraded (data not shown). Restoration of SNORD97 and SCARNA97 accumulations in *SNORD97* knockout and *SCARNA97* knockout cells abolished arsenite-induced 3′ tRF production, demonstrating that SNORD97 and SCARNA97 expression inhibits tRNA^Met^(CAT) cleavage at C34 (Fig. 5A, lanes 6,10). Further connecting angiogenin to in vivo tRNA^Met^(CAT) cleavage, administration of the angiogenin small molecule inhibitor N65828 to arsenite-treated *SNORD97 + SCARNA97* knockout cells obliterated 3′ tRF accumulation in a concentration-dependent manner (Fig. 5B, lanes 3,4; Kao et al. 2002; Blanco et al. 2014). Angiogenin is a secreted protein that can actively translocate into neighboring cells (Fett et al. 1985). Inclusion of recombinant angiogenin into the medium of noninduced *SNORD97 + SCARNA97* knockout cells promoted 3′ tRF accumulation, corroborating the conclusion that stress-induced cleavage of human tRNA^Met^(CAT) at C34 is catalyzed by angiogenin (Fig. 5B, lanes 5,6).

Ribose methylation of the wobble cytidine is an evolutionarily conserved feature of tRNA^Met^(CAT) and has been assumed to contribute to accurate tRNA decoding (Grosjean et al. 2010; Jackman and Alfonzo 2013; Boccaletto et al. 2018). However, as compared with the wild-type HAP1 cells, the *SNORD97 + SCARNA97* knockout cells showed no detectable growth and proliferation deficiencies, suggesting that 2′-O-methylation of the wobble cytidine of tRNA^Met^(CAT) has no major impact on translation accuracy and efficiency (Supplemental Fig. S7). Instead, an apparent role of Cm34 is in protection of mature tRNA^Met^(CAT) from angiogenin-mediated processing into a metabolically stable 3′ tRF that may function as a regulatory RNA. It is conceivable that under certain conditions, putative regulatory tRF production from tRNA^Met^(CAT) is controlled by partial 2′-O-methylation of C34 by SNORD97 and SCARNA97. Similar to Cm34 in tRNA^Met^(CAT), cytosine-5 methylations (m5C) at C34 and C38 can protect other tRNAs from angiogenin cleavage (see above). Mutations in the methyltransferases responsible for m5C34 and m5C38 synthesis in tRNAs result in tRF overaccumulation, cause neuro-developmental disorders in humans and mice, and impact mobile element expression and DNA repeat integrity in *Drosophila* (Blanco et al. 2014; Genenncher et al. 2018). Finally, in response to nutrient deprivation, the yeast U2 spliceosomal snRNA undergoes pseudouridylations at positions 56 and 93, catalyzed by the Pus7 pseudouridine synthase and the snR81 pseudouridylation guide snoRNP, respectively (Wu et al. 2011). Stress-induced pseudouridylation of U2 is believed to promote correct pre-mRNA splicing. Taken together, the above observations support the idea that posttranscriptional RNA modification, including sno/scaRNP-mediated 2′-O-methylation and pseudouridylation, can play important regulatory roles under certain physiological and stress conditions.

In summary, 2′-O-methylation of the wobble cytidine of tRNA^Met^(CAT) is the first eukaryotic RNA-guided RNA modification reaction that targets a cellular RNA other than rRNA and snRNA and uses both a nucleolar and a CB-specific guide RNA. Our results also point to a regulatory link between RNA-directed tRNA modification and angiogenin-mediated tRF processing.

## Materials and methods

### General procedures

DNA and RNA manipulations were performed according to standard laboratory procedures. Oligodeoxynucleotides were purchased from Eurofins MWG. Recombinant angiogenin and the small molecule angiogenin inhibitor N-65828 [8-amino-5-(4′-hydroxybiphenyl-4-ylazo)naphthalene-2-sulfonate] were kindly provided by Dr S. Blanco (Center for Cooperative Research in Biosciences, Derio, Spain).

### Expression plasmids

For construction of pGL/SNORD97 and pGL/SCARNA97 expression plasmids, the human *SNORD97* and *SCARNA97* genes were PCR-amplified and inserted into the ClaI and XhoI sites of the pGL expression vector using PCR-introduced ClaI and XhoI restriction sites. To obtain pGL/SCARNA97del, the T65–T154 internal fragment of the *SCARNA97* gene was eliminated from the pGL/SCARNA97 expression plasmid by a PCR-based approach. The pIRESpuro2/SNORD97 and pIRESpuro2/SCARNA97 expression constructs used for stable transformation of HAP1 cells were generated by PCR amplification of the globin-SNORD97- and globin-SCARNA97-coding regions of the pGL/SNORD97 and pGL/SCARNA97 plasmids and insertion of the resulting fragments into the EcoR1 and Not1 sites of the pIRESpuro2 expression plasmid (Addgene). The identity of each construct was confirmed by sequence analysis.

### CRISPR–Cas9 genome editing

Human HAP1 cells were cultured in Iscove's modified Dulbecco's medium (Gibco) supplemented with 10% fetal calf serum (PAN-Biotech), 100 U/mL penicillin (Sigma), and 1 mM sodium pyruvate (Gibco). Transfection of HAP1 cells with expression plasmids was performed by electroporation with Gene Pulser (Bio-Rad) at settings 270 V and 950 µF. *SNORD97* knockout and *SCARNA97* knockout cell lines were generated by transfection of HAP1 cells with recombinant pSpCAS9(BB)-2A-Puro (PX459) V2.O vectors (Addgene) expressing appropriately designed CRISPR guide RNAs targeting the *SNORD97* and *SCARNA97* intronic snoRNA genes (Fig. 2A). Single-cell-derived clonal cell lines were generated in the presence of 1.5 µg/mL puromycin, and the knockout phenotype of the selected cells was confirmed by genomic PCR and Northern blot analysis.

### RNA analyses

RNAs from HeLa and HAP1 cells were isolated by guanidinium thiocyanate-phenol-chloroform extraction. For Northern blotting, 10 µg of cellular RNAs was fractionated on 6% sequencing gels, electroblotted onto a Hybond-N nylon membrane (GE Healthcare), and hybridized with ^32^P-labeled sequence-specific oligodeoxynucleotide probes (sequences are available on request).

### Primer extension mapping of 2′-O-methylated nucleotides

Total cellular RNAs (20 µg) were partially hydrolyzed in 20 µL of 80% formamide containing 0.4 mM MgCl_2_ at 100°C. Samples were collected after 5, 10, 15, and 20 min of incubation, and RNAs were precipitated with ethanol and used as templates for primer extension analysis with the SuperScript II reverse transcriptase as recommended by the manufacturer (Thermo Fisher Scientific).

### FISH microscopy

Oligodeoxynucleotides labeled with cyanine 3 (Cy3) (Eurofins Genomics) and complementary to SNORD97 C*GTCAAGAACTCCAGCATAAAGTCCTCTTAA* or SCARNA97 T*CAGATCTCATAATCTTACCCTTCAC* (asterisks indicate Cy3 positions) were used as probes for in situ hybridization in 2× SSC containing 15% formamide (Darzacq et al. 2002). Nucleoli were detected by transient expression of GFP-fibrillarin (Darzacq et al. 2002), and Cajal bodies were stained with anticoilin antibody (1:100 dilution; Abcam). Slides were mounted in 90% glycerol, 1× PBS, 0.1 µg/mL DAPI, and 1 mg/mL *p*-phenylenediamin. Raw images were acquired using a Hamamatsu ORCA-Flash 4.0 camera (CMOS Scientific) mounted on an inverted IX-81 microscope (Olympus) with an UPlan SApo ×100 1.4 oil objective. Final images were prepared with Adobe Photoshop.

## Supplementary Material

Supplemental Material
