# Supplementary material for: Cooperative 2′-O-methylation of the wobble cytidine of human elongator tRNAMet(CAT) by a nucleolar and a Cajal body-specific box C/D RNP
Source: Genes Dev. 2019 Jul 1;33(13-14):741–6. doi: 10.1101/gad.326363.119 (PMC6601510; doi:10.1101/gad.326363.119)
Supplement: Supplemental Material [file supp_33_13-14_741__index.html]

Cooperative 2′-O-methylation of the wobble cytidine of human elongator tRNAMet(CAT) by a nucleolar and a Cajal body-specific box C/D RNP — Supplemental Material 

# Cooperative 2′-O-methylation of the wobble cytidine of human elongator tRNAMet(CAT) by a nucleolar and a Cajal body-specific box C/D RNP

## Supplemental Material

- Supplemental\_material.pdf
