# Supplementary material for: Cooperative 2′-O-methylation of the wobble cytidine of human elongator tRNAMet(CAT) by a nucleolar and a Cajal body-specific box C/D RNP
Source: Genes Dev. 2019 Jul 1;33(13-14):741–6. doi: 10.1101/gad.326363.119 (PMC6601510; doi:10.1101/gad.326363.119)
Supplement: Supplemental Material [file supp_gad.326363.119_Supplemental_material.pdf]

Supplemental Material

Cooperative 2'-O-methylation of the wobble cytidine of human elongator tRNA^{Met}(CAT) by a nucleolar and a Cajal body-specific C/D RNP

Patrice Vitali and Tamás Kiss

Supplemental Figures

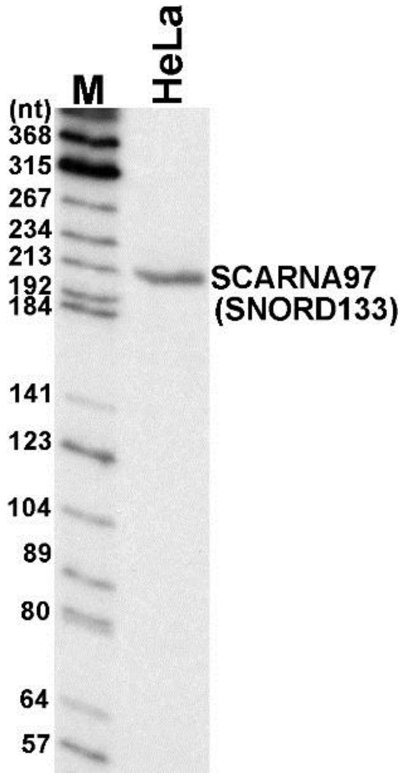

Supplemental Fig. S1. Northern blot analysis of the expression of human SCARNA97 (formerly called SNORD133). HeLa total cellular RNAs (10 µg) were size-fractionated on a 6% denaturing polyacrylamide gel, electroblotted onto a nylon membrane and probed with a SCARNA97-specific terminally ^{32}P-labelled oligodeoxynucleotide. Lane M, DNA size markers in nucleotides (nt).

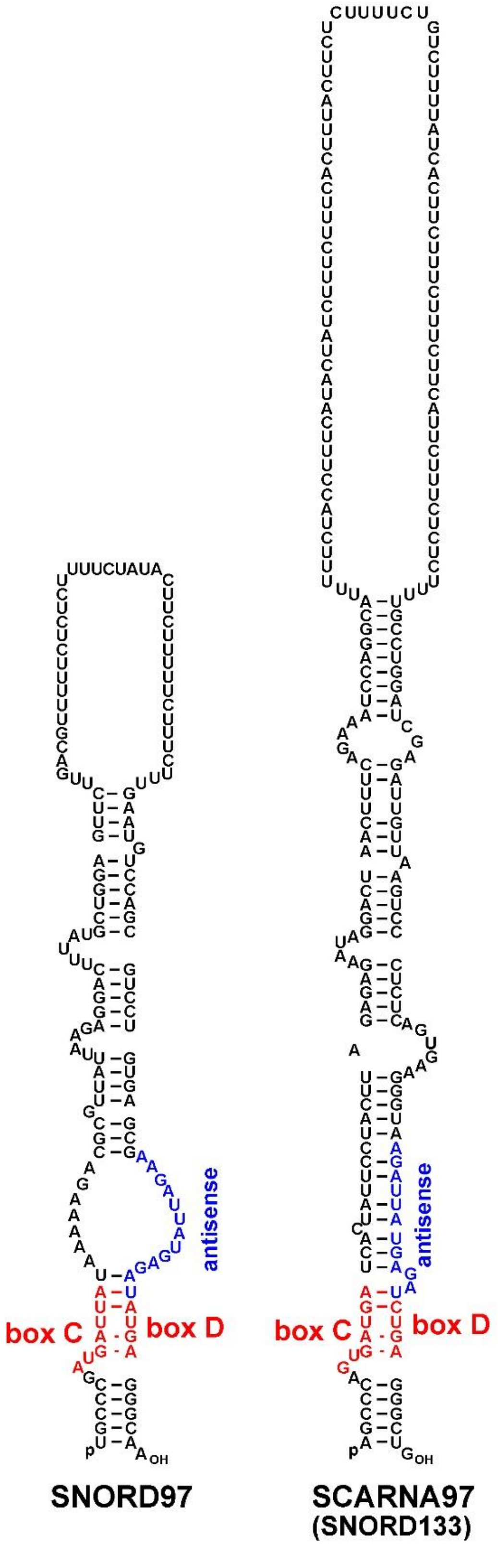

Supplemental Fig. S2. Predicted secondary structures of human SNORD97 and SCARNA97 box C/D RNAs. The conserved C and D boxes (red) and the antisense elements (blue) are highlighted. Due to their shortage of purine residues, the internal pyrimidine-rich regions are unable to fold into conventional secondary structures supported by Watson-Crick base-pairings.

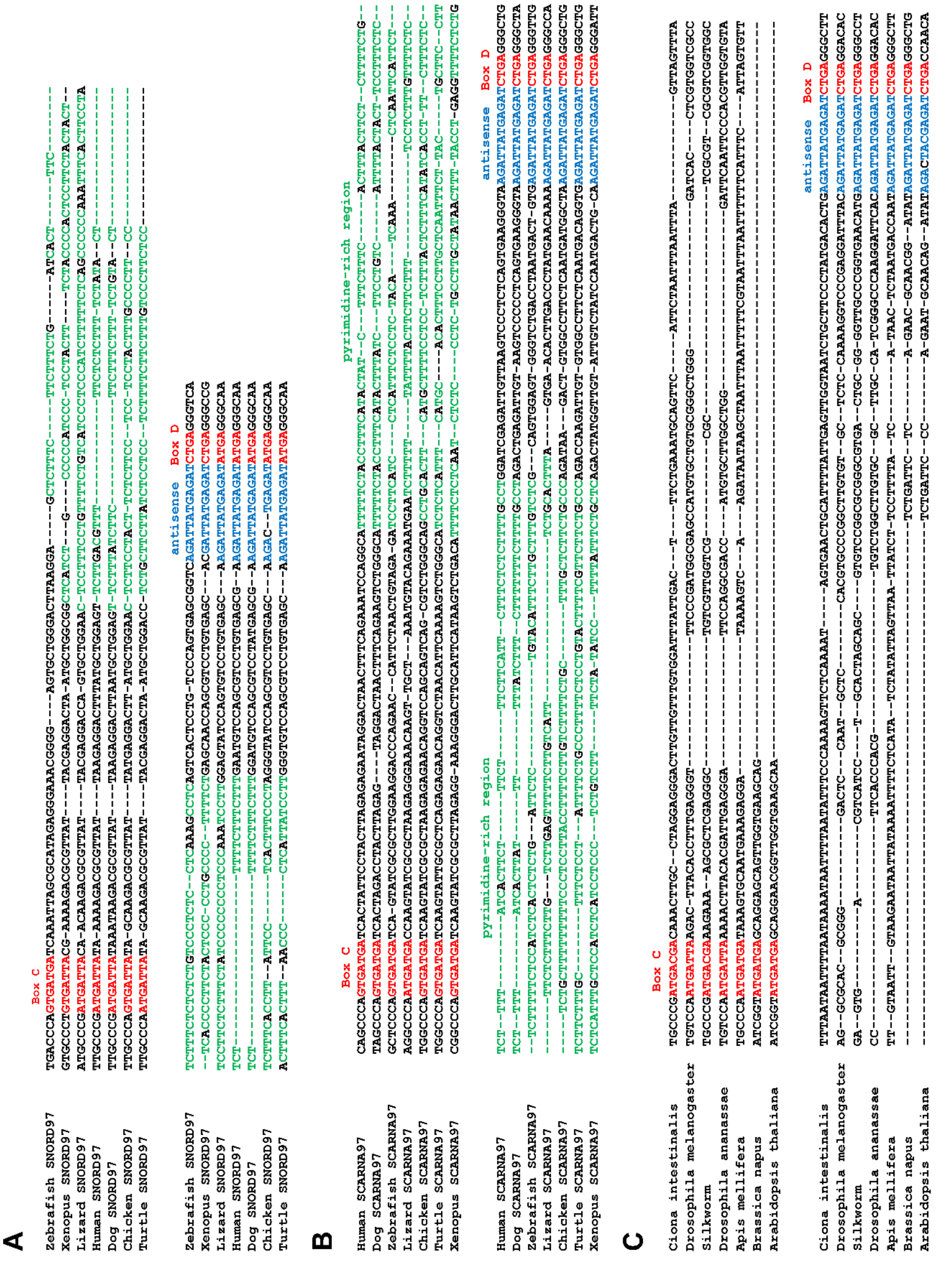

Supplemental Fig. S3. Alignments of predicted vertebrate SNORD97 (*A*) and SCARNA97 (*B*) and non-vertebrate SNORD97/SCARNA97 gene sequences. The conserved box C and D sequences are in red and the predicted antisense elements are in blue. Pyrimidine residues in the internal regions of vertebrate SCARNA97 and SNORD97 are in green. Vertebrate SNORD97 and SCARNA97 genes likely originated from a gene duplication event at the early stage of vertebrate evolution. Supporting this idea, in fish, both SNORD97 and SCARNA97 are encoded within introns of the LARP4 gene and the SCARNA97 gene appears in the EIF4G2 gene only in amphibians.

```
human1        GCCCTC-TTAGCGCAGCTGGC-AGCGCGTCAGTCTCATAATCTGAA-GGTCCTGAGTTCAAGCCTCAGAGAGGGCA
human2        GCCCTC-TTAGCGCAGCGGGC-AGCGCGTCAGTCTCATAATCTGAA-GGTCCTGAGTTCGAGCCTCAGAGAGGGCA
human3        GCCCTC-TTAGCGCAGCGGGC-AGCGCGTCAGTCTCATAATCTGAA-GGTCCTGAGTTCGAGCCTCAGAGAGGGCA
human4        GCCCTC-TTAGCGCAGCGGGC-AGCGCGTCAGTCTCATAATCTGAA-GGTCCTGAGTTCGAGCCTCAGAGAGGGCA
human5        GCCCTC-TTAGCGCAGCGGGC-AGCGCGTCAGTCTCATAATCTGAA-GGTCCTGAGTTCGAGCCTCAGAGAGGGCA
human6        GCCTCC-TTAGCGCAGTAGGC-AGCGCGTCAGTCTCATAATCTGAA-GGTCCTGAGTTCGAACCTCAGAGGGGGCA
human7        GCCTCC-TTAGCGCAGTAGGC-AGCGCGTCAGTCTCATAATCTGAA-GGTCCTGAGTTCGAACCTCAGAGGGGGCA
human8        GCCCTC-TTAGCGCAGTGGGC-AGCGCGTCAGTCTCATAATCTGAA-GGTCCTGAGTTCGAGCCTCAGAGAGGGCA
human9        GCCTCG-TTAGCGCAGTAGGT-AGCGCGTCAGTCTCATAATCTGAA-GGTCGTGAGTTCGATCCTCACACGGGGCA
Dog           GCCTTC-TTAGCGCAGTGGGC-AGCGCGTCAGTCTCATAATCTGAA-GGTCCTGAGTTCGAGCCTCAGAGAGGGCA
Chicken       GCCCTC-TTAGCGCAGCAGGC-AGCGCGTCAGTCTCATAATCTGAA-GGTCCTGAGTTCGAGCCTCAGAGAGGGCA
Lizard        GCCTCGT-TGGCGCAGTAGGC-AGCGCGTCAGTCTCATAATCTGAA-GGTCGTGAGTTCGAGCCTCACACGGGGCA
Turtle        GCCTCG-TTAGCGCAGTAGGT-AGCGCGTCAGTCTCATAATCTGAA-GGTCGTGAGTTCGATCCTCACACGGGGCA
Xenopus       GCCTCG-TTAGCGCAGTAGGT-AGCGCGTCAGTCTCATAATCTGAA-GGTCGTGAGTTCGATCCTCACACGGGGCA
Zebrafish     GCCTCGT-TGGCGCAGTAGGC-AGCGCGTCAGTCTCATAATCTGAA-GGTCGTGAGTTCGAGCCTCACACGGGGCA
Ciona         GCCTCG-ATGGCGCAGTAGGC-AGCGCGTCAGTCTCATAATCTGAA-GGTCGTGAGTTCGATCCTCACTCGGGGCA
Drosophila    GCCTCGA-TGGCGCAGTTGGC-AGCGCGTAAGTCTCATAATCTTAA-GGTCGTGAGTTCGAGCCTCACTCGGGGCA
Bombyx        GCCTCGTGTGGCGCAGTATGC-AGCGCGTAAGTCTCATAATCTTAAGTGTCGTGAGTTCGATCCTCACCCGGGGCA
Arabidopsis   GG-GGTGGTGGCGCAGTTGGCTAGCGCGTAGGTCTCATAATCCTGA-GGTCGAGAGTTCGAGCCTCTCTCACCCCA
Brassica      GG-GGTGGTGGCGCAGTTGGCTAGCGCGTAGGTCTCATAATCCTGA-GGTCGAGAGTTCGAGCCTCTCTCACCCCA
              *       * ******   *  *******  ***********   * ****  ****** * ****        **
```

Supplemental Fig. S4. Alignment of predicted vertebrate and non-vertebrate tRNA^{Met}(CAT) gene sequences. Evolutionarily invariant nucleotides are marked by asterisks. Sequences complementary to the conserved antisense elements of SNORD97 and SCARNA97 are in blue. The CAT anticodon sequences are shaded and the 2'-O-methylated C34 wobble residues are in red.

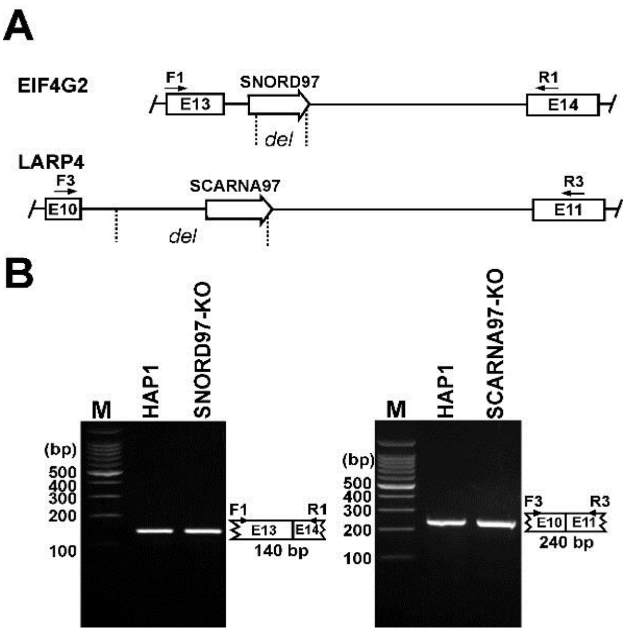

Supplemental Fig. S5. Deletion of the SNORD97 and SCARNA97 intronic RNA genes has no effect on the splicing of the EIF4G2 and LARP4 host pre-mRNAs. (*A*) Schematic structures of the relevant regions of human EIF4G2 and LARP4 genes and positions of the forward (F1 and F3) and reverse (R1 and R3) primers used for RT-PCR are shown. The borders of genomic deletions (*del*) are indicated by dashed lines. (*B*) RT-PCR analysis of the expression of EIF4G2 and LARP4 mRNAs in HAP1, SNORD97-KO and SCARNA97-KO cells. The structures and expected lengths of the amplified EIF4G2 and LARP4 cDNA fragments are shown. Lanes M, size markers in base-pairs (bp).

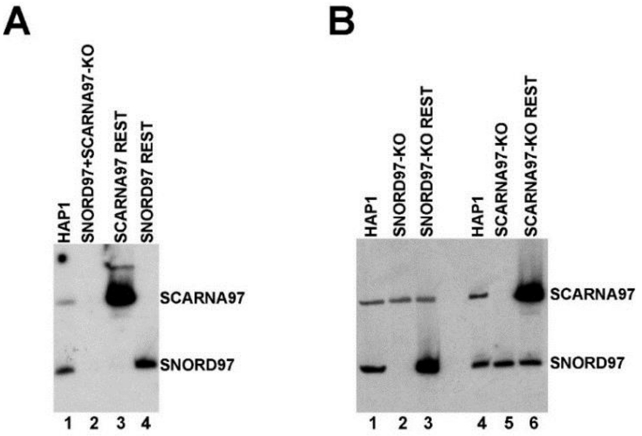

Supplemental Fig. S6. Northern blot analysis of the accumulation of endogenous and ectopically expressed SCARNA97 and SNORD97. (*A*) Restoration of the expression of SCARNA97 (lane 3) or SNORD97 (lane 4) in SNORD97+SCARNA97-KO cells (lane 2). (*B*) Restoration of SNORD97 (lane 3) and SCARNA97 (lane 6) accumulation in SNORD97-KO (lane 2) and SCARNA97-KO (lane 5) cells. Control hybridization of wild-type HAP1 RNAs is shown (lanes 1 and 4). Exogenously expressed SNORD97 and SCARNA97 accumulated in the nucleoli and CBs, respectively (see Fig. 1B).

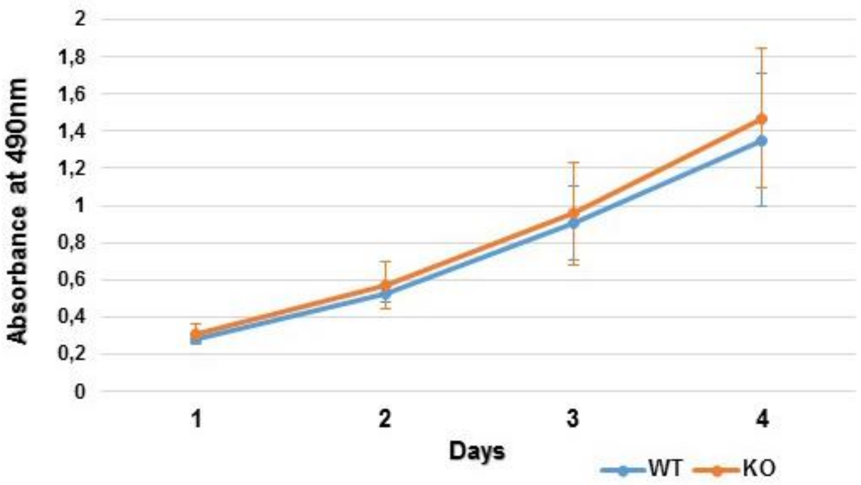

Supplemental Fig. S7. Comparison of the proliferation rates of human HAP1 (WT) and SNORD97+SCARNA97-KO (KO) cells. The MTS-based CellTiter 96® AQ_{ueous} One Solution Cell Proliferation Assay was used to monitor cell proliferation according to the manufacturer's instructions (Promega). Equal numbers (5×10^3) of HAP1 and SNORD97+SCARNA97-KO cells were distributed to the wells of a 96-well plate in Iscove's Modified Dulbecco's medium supplemented with 10% fetal calf serum. Cells were incubated under standard culture conditions for 1, 2, 3 and 4 days. One hour before measuring absorbance at 490 nm by an ELISA plate reader, 20 µl of CellTiter 96® AQ_{ueous} One Solution Cell Proliferation Reagent was added to each well. Each point indicates the mean of ± SD of 3 replicas.
